# Estimation of Femoral Neck Anteversion in Adults: A Comparison Between Clinical Method, Radiography, and Computed Tomography at a Tertiary-care Center in Eastern India

**DOI:** 10.7759/cureus.4469

**Published:** 2019-04-16

**Authors:** Bishnu P Patro, Sudarsan Behera, Sudhanshu S Das, Gurudip Das, Saroj K Patra, Vinay Prabhat

**Affiliations:** 1 Orthopaedics, All India Institute of Medical Sciences, Bhubaneswar, IND; 2 Trauma and Emergency, All India Institute of Medical Sciences, Bhubaneswar, IND

**Keywords:** anteversion, femur neck, magilligan method, computed tomography

## Abstract

Introduction

Femoral anteversion is the anterior inclination of the femoral neck and head in relation to the shaft of the femur. Femoral anteversion provides torsional stability of the hip - an important clinical factor for conditions such as trauma, arthroplasty, developmental dysplasia of the hip, and Legg-Calve Perthes disease. Precise measurement is important to avoid instability in pathological conditions of the hip. Computed tomography (CT) measures the angle more accurately as compared to plain radiography and is considered the gold standard procedure for measurement. Patients are exposed to significantly more ionizing radiation in CT, especially the pediatric population, which is more susceptible.

Material and methods

A prospective study of 25 individuals was undertaken wherein the femoral anteversion angle was comparatively measured by clinical, radiographic, and CT methods.

Results

The radiological evaluation depicted mean values that were far from those of the CT evaluation as compared to the clinical evaluation.

Conclusion

The clinical method (trochanter prominence angle test) can be used to measure femoral anteversion to avoid exposure to ionizing radiation and cases where CT is unavailable.

## Introduction

Femoral anteversion was first described by Wolff in 1868, as the anterior inclination of the femoral neck and head in relation to the shaft of the femur. In other words, femoral anteversion is the relationship of the axis of the femoral neck to the transcondylar axis or coronal axis of the distal femur. The angle of anteversion in an individual with a mature skeleton is 10%-15% [1-3]. However, it is intraracial and intraindividual, that is, in the contralateral side of an individual, variations have been reported [1-3].

Normally, femoral anteversion ensures torsional stability at the hip joint. Any change in the version leads to secondary changes in the hip and alters joint stability. Femoral version is an important clinical factor in pathological conditions, such as developmental dysplasia of the hip, hip joint arthroplasty, Legg-Calve-Perthes disease, slipped capital femoral epiphysis, hip stability in patients with cerebral palsy, and sequelae of femoral fractures [1-10].

The angle of femoral anteversion in a particular population should be documented by a method that is accurate, easily available, reproducible, and consistent. The accurate estimation of femoral neck anteversion in living subjects is associated with considerable difficulty as well as many shortcomings and a lack of replicability. The estimation of the anteversion angle on the dry bone is considered to be the most accurate method [11]; however, the greatest drawback with this method is the inclusion of the femur from some skeletons with pathologic conditions and an unknown influence on the final outcome. Nevertheless, the measurement of the dried femur provides a femoral anteversion profile of the sample population although it may not be relevant for clinical practice because the clinical measurement of the femoral anteversion may differ from those obtained on the dry femur [11].

Femoral anteversion has been determined by different investigators through different methods, such as the clinical, radiological, ultrasonographic, computed tomographic, and magnetic resonance imaging modalities [12-18]. Netter first described the clinical evaluation of femoral anteversion by the trochanteric prominence test. The clinical method of evaluation of femoral anteversion is influenced by various extrinsic and intrinsic variables, such as tension of the hip capsule, inclination of the acetabulum, muscle and fat mass over the trochanter, and patient cooperation; therefore, this method is often not used for investigative purposes. Biplanar orthogonal radiography is routinely used to measure the angle of anteversion but is often inadequate for surgical planning. Computed tomography (CT) measures the angle more accurately as compared to plain radiography and is considered the gold standard procedure for the measurement of femoral anteversion [17-19]. Patients, especially the pediatric population, which is more susceptible to adverse effects, are exposed to significantly more ionizing radiation in CT [20]. Therefore, the frequent use of CT for the measurement of anteversion is questionable.

In eastern India, where external rotation and abduction is more common when compared to the population in western India, no study has compared the measurement of femoral anteversion by clinical, radiographic, and CT methods. We prospectively studied 25 individuals to ascertain the correlation between CT and clinical method and radiography and evaluated which method would correlate better with the angle measured by CT, with the aim to avoid radiation hazards and measure the angle when planning surgery.

## Materials and methods

From February 2004 to November 2005, we selected 25 individuals of either sex in the age group of 40 to 60 years from among patients or the attendants accompanying patients who presented to the orthopedic outpatient department for ailments other than those affecting the lower limb. Individuals with any sort of deformity of the lower limb, a history of surgery around the hip joint or neuromuscular paralytic disorders, and osteopenic states, such as osteomalacia and malignancy, were excluded from this prospective study. Informed consent was obtained and an estimation of the anteversion was evaluated by: a) clinical; b) radiographic; and c) computed tomographic methods.

Clinical evaluation

**Figure 1:**
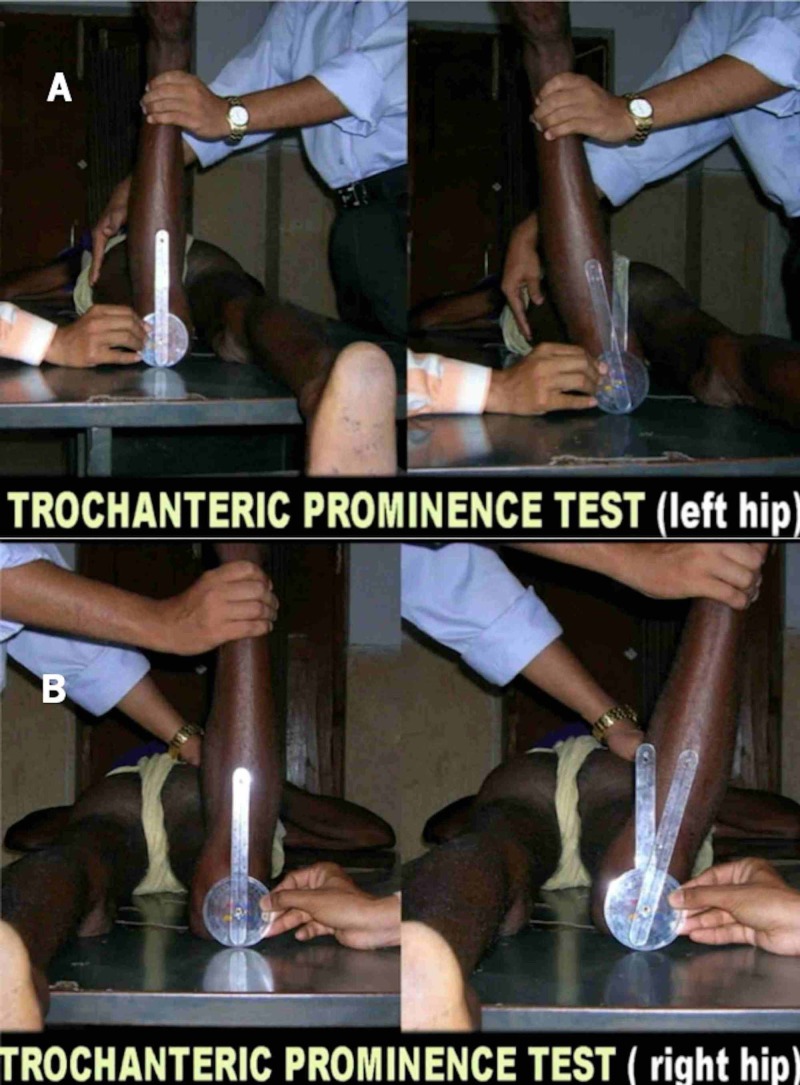
Trochanteric prominence angle test A: left hip B: right hip

Figures 1A-1B present the details of the clinical evaluation of anteversion.

Individuals were instructed to lie prone on a hard couch. When the version of the right hip was measured, the examiner stood on the contralateral side of the patient with the knee flexed to 90°, the examiner’s left hand was used to palpate the greater trochanter, while the right-hand internally rotated the hip by bending the leg outward. The most lateral position of the greater trochanter was represented by the point of maximum trochanteric prominence while the neck of the femur was presumed to be parallel to the floor. The angle subtended between the tibia and true vertical was measured with the help of a goniometer at a maximum prominence of the greater trochanter and was noted as an angle of anteversion. It was repeated three times by two examiners to eliminate the bias as far as possible.

Radiographic evaluation

We undertook radiographic evaluation according to the procedure described by Magilligan [15]; roentgenographic examination in two planes was used routinely to determine the relationship of the femoral head and neck to the acetabulum and the shaft of the femur. Patients lay supine on the x-ray table with both hips abducted at 100-150 and the x-ray tube positioned between their thighs. An anteroposterior (AP) view of the head of the femur was taken with the x-ray tube centered over the hip joint such that the femur was parallel to table and in a neutral rotation - that is, the dicondylar plane was parallel to the table. On AP view of the hip joint, the long axis of the femoral neck was estimated. Thereafter, a horizontal roentgenogram of the hip was obtained, with the cassette held against the lateral side of the thigh and trunk, and the head of the X-ray tube was placed between the thighs at the desired level. The cassette was held perpendicular to both the table and the dicondylar plane and parallel to the long axis of the femoral neck that was estimated from the AP view of the hip joint without changing the position of the subject. The axis of the femoral neck was the line that passed through the center of the neck and bisected the cortical borders at the base and sub-capital region of the neck of the femur. The axis of the femoral shaft was the line that passed through the central axis of the shaft and bisected the cortical borders of the proximal three cm of the femoral shaft. The acute cervicofemoral angles thus revealed on the AP and lateral radiograms were designated alpha (a) and beta (b), respectively. After a and b were calculated, the true angle of femoral anteversion, q, was obtained by a simple trigonometric formula used by Magilligan.

**Figure 2:**
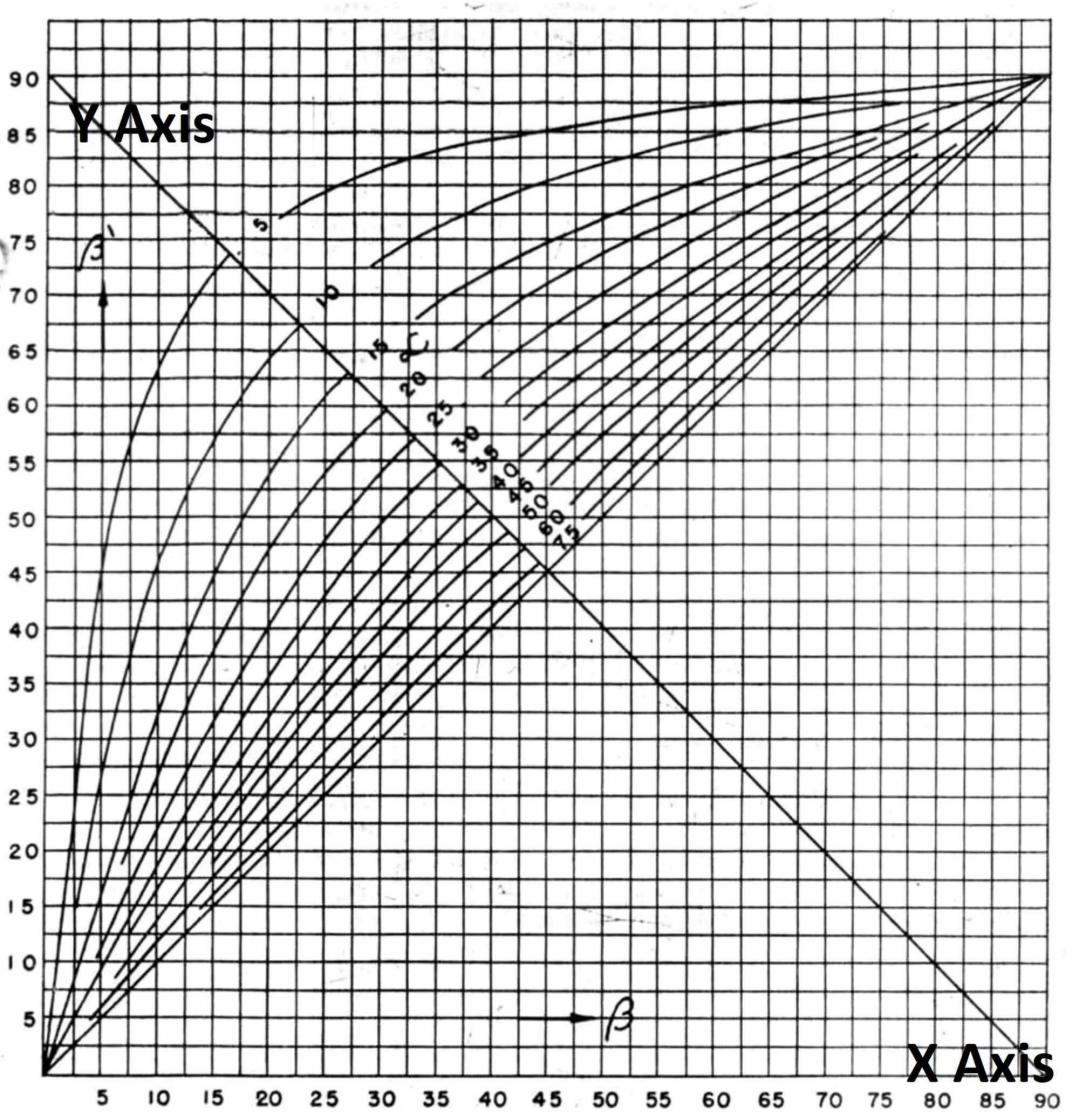
Graphical evaluation of true femoral anteversion from angle alpha and angle beta X-axis: angle beta Y-axis: angle alpha

Alternatively, the true angle of anteversion, q, could be calculated with an easy reference graph (Figure 2 and Table 1).

**Table 1:** Easy reference table for the determination of angle of anteversion from the cervicofemoral angle in the anteroposterior (Alpha) and lateral (beta) views as per Magilligan

b/a	80	70	60	50	45	40	30	20	10
10	10	11	12	13	14	15	19	27	46
20	20	21	23	25	27	29	36	47	64
25	25	26	27	31	33	36	43	53	69
30	30	31	34	37	39	42	49	60	73
35	35	36	39	42	45	48	55	64	76
40	41	42	44	48	50	52	59	68	78
45	46	47	49	52	55	57	64	71	80
50	51	52	54	57	59	62	67	74	82
55	55	56	59	62	63	66	70	76	83

Table 1 presents a more lucid and accessible option as compared to that in the graph. However, in both the graph and the table, by taking any value of a or b, both from 0 to 900, the possible angle of q can be calculated from any combination of a and b within the specified range.

CT evaluation

**Figure 3:**
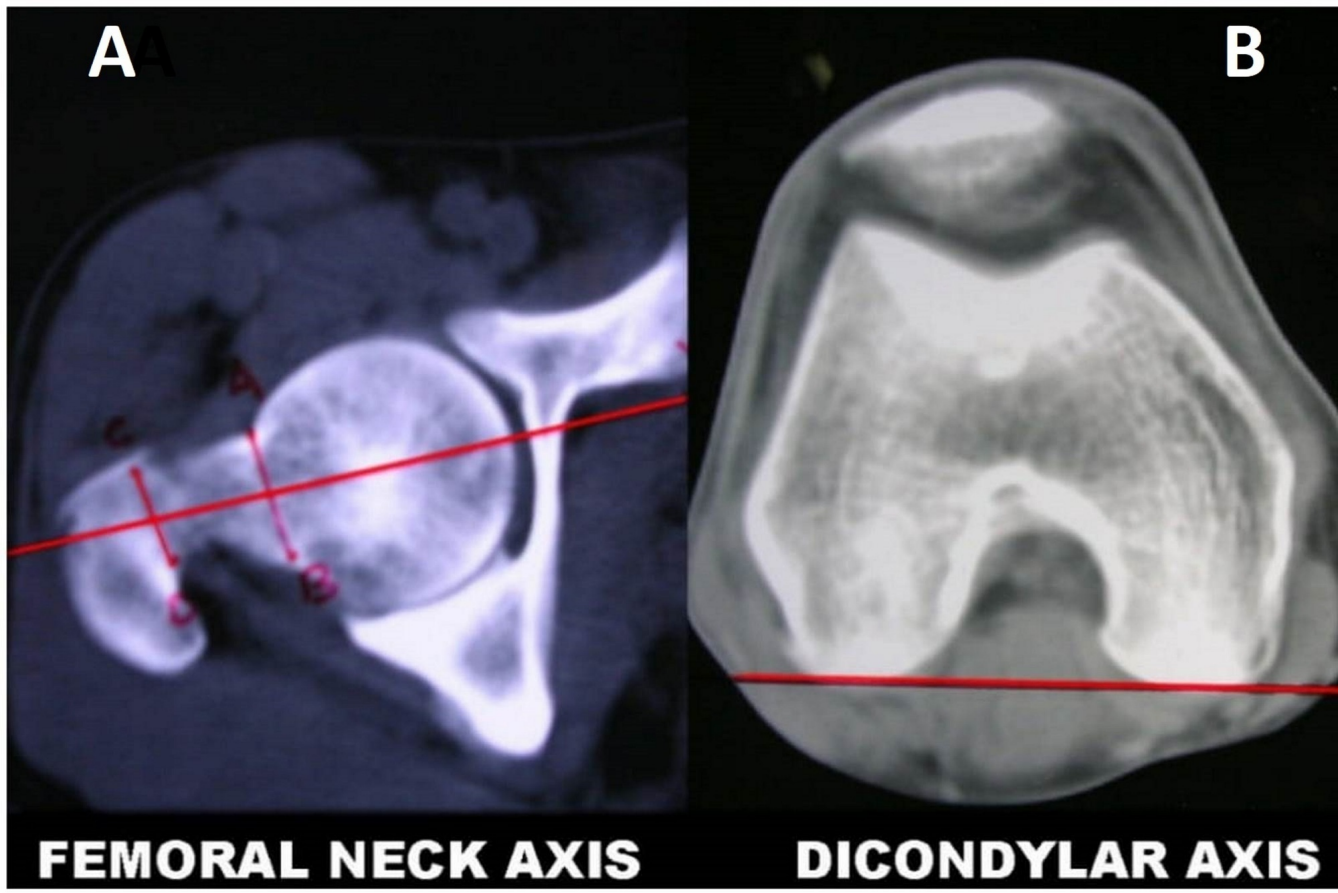
Computed tomographic evaluation A: Femoral neck axis B: Dicondylar axis

For this, the subject lay supine on the CT table with his/her legs secured to the table with Velcro straps to prevent movement at the upper and lower end of the femur during and between the scans. A series of tomographic cross-sectional sections were taken at the hip and knee levels. These CT sections were reconstructed with a special inbuilt programmer. From all the images obtained, we selected one each that clearly depicted the center of the head and the neck length of the femur, usually at the upper border of the trochanter, and showed the outline of the femoral condyles at the lower end of the femur. The axis of the femoral neck was determined by a line that connected two points at the base and sub-capital region of the femoral neck and equidistant between the superior and inferior surfaces of the femoral neck (Figure 3A). A line connecting two points along the most posterior aspect of the femoral condyles, that is, the dicondylar axis, was selected for the measurement of the axis (Figure 3B).

**Figure 4:**
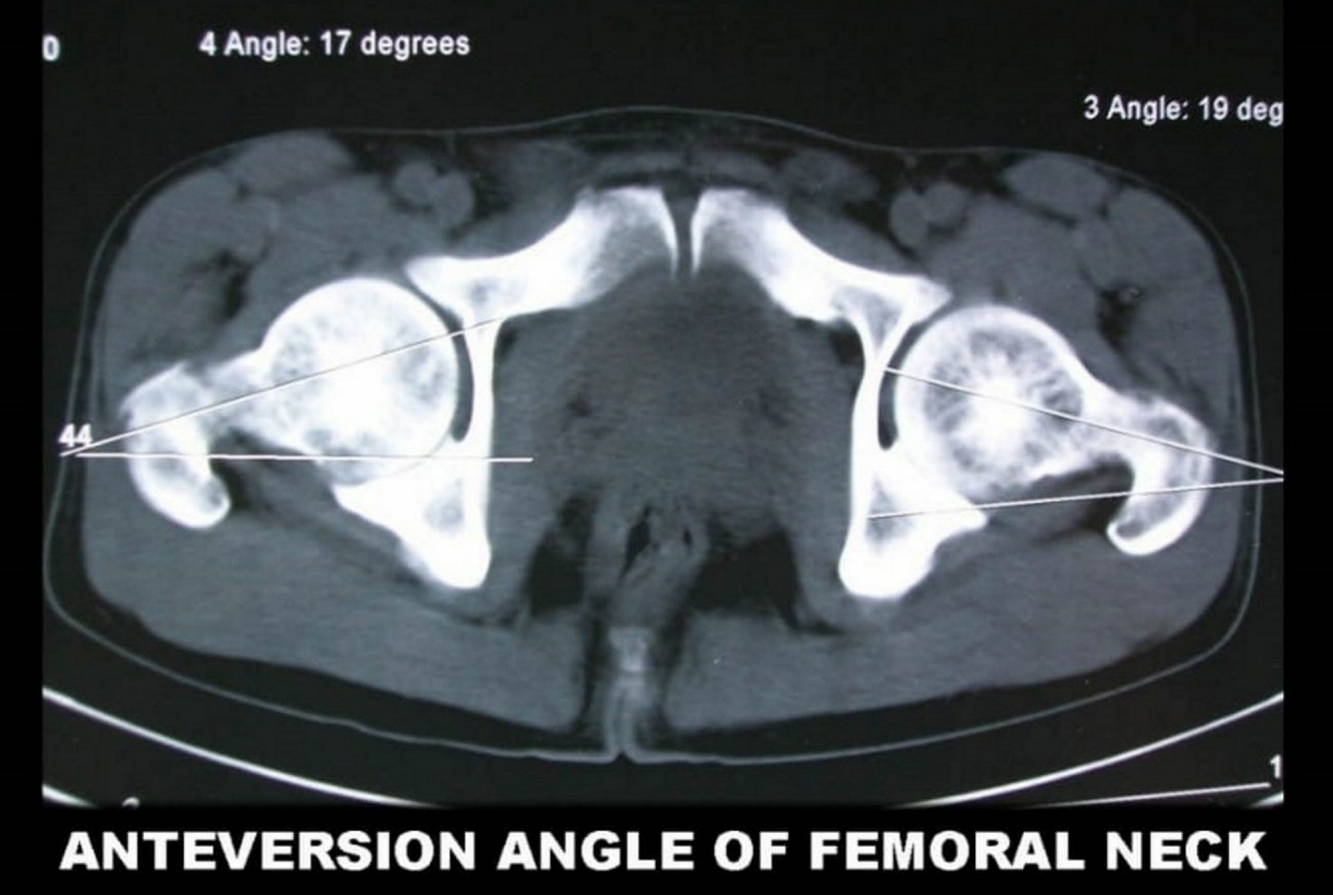
Anteversion angle of femoral axis calculated from computed tomography

After the axis at the femoral neck and the dicondylar axis were evaluated, the images were superimposed and the angle between them was measured directly using an inbuilt option of the CT scanner (Figure 4).

The mean, median, and standard deviation of the femoral anteversion (FAV) angle were measured with the Statistical Package for Social Sciences (SPSS) version 17.0 (IBM Corp., Armonk, NY, US). Variations between the values of the FAV angle, due to the subjects, methods applied, or unexplained factors, were analyzed by the analysis of variance (ANOVA). To identify differences between individual methods, we applied multiple comparison tests. The least significant difference test was applied to evaluate the efficacy of each method.

## Results

Of the 25 individuals examined, 19 were male and six were female. The femoral anteversion angles (FAV) obtained by different methods were evaluated with reference to the parameters of age, sex, side, height, and weight. The age ranged from 40 to 60 years, with most subjects (60%) falling into the 51-60 years bracket. The male:female ratio was 3.16:1. Fourteen subjects weighed less than 60 kg, and 11 weighed more than 60 kg.

With CT evaluation as the gold standard in this study, a comparison of the mean values from different methods showed that the values obtained by the clinical method were nearer to those from the CT evaluation as compared to those from the radiological evaluation (Table 2).

**Table 2:** Summary of the measurements of the anteversion angle from the CT, radiography, and clinical evaluation methods Abbreviations: CT-L: Computed Tomography Anteversion Left Hip, CT-R: Computed Tomography Anteversion Right Hip, XR-L: Radiographic Measurement of Anteversion Left Hip, XR-R: Radiographic Measurement of Anteversion Right Hip, CL:A-L: Clinical Measurement of Anteversion Left Hip by Evaluator A, CL:B-L: Clinical measurement of Anteversion Left Hip by Evaluator B, CL:A-R: Clinical Measurement of Anteversion Right Hip by Evaluator A, CL:B-R: Clinical Measurement of Anteversion Right Hip by Evaluator B, n: Number of Subjects, SD: Standard Deviation, SE: Standard Error, LCL: Lower Confidential Limit, UCL: Upper Confidential Limit, CI: Confidence Interval

	CT-L	CT-R	XR-L	XR-R	CL:A-L	CL:B-L	CL:A-R	CL:B-R
n	25	25	25	25	25	25	25	25
Mean	12.08	12.24	19.16	19.32	15.56	15.36	15.76	15.92
SD	4.28	5.11	4.98	5.54	4.85	4.68	4.47	5.51
SE	0.86	1.02	1.00	1.11	0.97	0.94	0.89	1.10
Max	22	25	37	36	29	30	30	36
Min	6	4	13	11	8	10	9	10
Range	16	21	24	25	21	20	21	26
LCL (95% CI)	10.31	10.13	17.10	17.03	13.56	13.43	13.91	13.65
UCL (95% CI)	13.85	14.35	21.22	21.61	17.56	17.29	17.61	18.19

There were no significant differences in the FAV angle between males and females that were evaluated by different methods (Table 3).

**Table 3:** Comparison of male and female anteversion angles measured with the CT, radiography, and clinical evaluation methods

	CT-L	CT-R	XR-L	XR-R	CL:A-L	CL:B-L	CL:A-R	CL:B-R
t-value	1.39	0.96	2.00	1.17	0.65	1.51	0.12	1.41
p-value	>0.05	>0.05	>0.05	>0.05	>0.05	>0.05	>0.05	>0.05
Significance	Not significant	Not significant	Not significant	Not significant	Not significant	Not significant	Not significant	Not significant

Variations in the FAV due to subjects, the methods applied, or unexplained factors were analyzed by ANOVA (Table 4).

**Table 4:** Analysis of variance test for both hips Note: SS, sum of square deviation; df, degree of freedom; MS, mean of spuare deviation; F, F-value; *p*, probability; Crit: P, critical – *P*. ***, indicates significance.

					Left side													Right side					
Source of variation					SS			df	MS			F			P		Crit: P	SS	df	MS	F	P	Crit:P
Between methods					627.72			3	209.24			18.99			<0.001***		6.04	626.99	3	209.00	25.98	<0.001***	6.04
Between subjects					1331.84			24	55.49			5.04			<0.001***		2.59	1991.14	24	82.96	10.31	<0.001***	2.59
Unexplained error					793.28			72	11.02									579.26	72	8.05			
Total					2752.84			99										3197.99	99				
	Left side							Right Side														
Source of variation	SS(Sum of spuare deviation)	Df(Degree of freedom)	MS(Mean of spuare deviation)	F(F-value)	P(Probability)	Sig.	Crit: P(Critical –P)	SS	df	MS	F	p	Sig	Crit:P
Between methods	627.72	3	209.24	18.99	<0.001	***	6.04	626.99	3	209.00	25.98	<0.001	***	6.04
Between subjects	1331.84	24	55.49	5.04	<0.001	***	2.59	1991.14	24	82.96	10.31	<0.001	***	2.59
Unexplained error	793.28	72	11.02					579.26	72	8.05				
Total	2752.84	99						3197.99	99													

The subjective variation was high (p<0.001); however, this is an aspect that cannot be eliminated in biological studies because of inter-individual variations in the pathophysio-morphology. There was a significant difference (p<0.001) between the three methods used for the determination of the FAV angle, although we could not identify the method that was erroneous. Multiple comparisons were undertaken using the LSD method, and a significant difference was found between the clinical and radiological methods compared to that of the CT method. The study could neither explain the accuracy of the clinical or radiological method nor validate the significance of a particular test in comparison to that of CT evaluation. Therefore, the diagnostic value was calculated by estimating the sensitivity, positive predictive value, and overall efficacy of each method by a comparison of the clinical and radiological findings with ±25% of the values from the CT evaluation. Less than 50% of values obtained by clinical or radiological evaluation were within ±25% of the values from the CT evaluation. However, when compared to that from the radiological evaluation, more values from the clinical evaluation group were within the ±25% range than those of the CT evaluation.

One interesting finding that may be incidental was that more values of the left hip evaluated by clinical or radiological evaluation were within the ±25% of those from the CT evaluation. Through clinical evaluation, it was explained that the unfamiliar position of the examiner to that of the subject, when examining the FAV of the right hip could be a cause. However, the variation between the left and right hips obtained by radiological findings could not be explained and was attributed to be an unexplained error.

A comparison of the values obtained by two separate individuals who used the clinical methods showed no significant differences in the outcome. Thus, the interobserver variation was found to be negligible.

## Discussion

Precise measurement of femoral neck anteversion is important for decision-making, surgical planning, and better outcome in the clinical management of conditions such as malalignment, torsional syndrome, developmental dysplasia of the hip, joint reconstruction, and trauma. An incorrect assessment of femoral anteversion could lead to altered gait, hip instability, rotational instability, and the development of secondary arthritis of the hip. Several methods such as clinical, radiological ultrasonographic, CT, and magnetic resonance imaging, have been used for the determination of femoral anteversion by different investigators [12-18]. 

In the above study, the FAV was analyzed by three different methods - clinical, radiographic, and CT; the values obtained were compared with the CT method as the gold standard by Lausten [19]. With a sensitivity of approximately 40%, the clinical evaluation method was nearly as accurate as the CT evaluation. Moreover, the interobserver variation was not significant (p>0.05). Although David highlighted the importance and superiority of the biplane method compared to that of axial tomography and fluoroscopic evaluation, Magilligan evaluated the FAV using the normal AP and lateral X-ray views of the hip joint thereby obviating the need for a special procedure and positioning through the use of a simple trigonometric formula with acceptable accuracy [10,14-15]. We observed that the radiological evaluation (Magilligan method), with a sensitivity of approximately 12%, was less accurate than the clinical evaluation. Similar findings were observed by Jain et al. and Ruwe et al., that is, that clinical evaluation is better than the radiological method (the Ogata method was used by Jain et al. and the Magilligan method by Ruwe et al.) [11-13]. Values derived for the left hip were more accurate than those for the right when evaluated radiologically, and we found this could be an incidental finding.

In this research work, CT was considered the gold standard. CT sections of the upper border of the greater trochanter were considered for the measurement of the axis of the femoral neck because it shows the maximum length of the neck of the femur. We did not consider the center of the femoral head for the calculation of the axis of the femoral neck because Kingsley found that the head of the femur is not centered on the neck in their study [21]. The identification of the femoral condylar plane is necessary for the measurement of anteversion. However, different condylar axes have been defined, for example, the classical table top, widest diameter, centroid, and bisector methods. Among the four methods of calculation, the table top, that is, the line passing through the posterior peak of condyles, was most reproducible and simple, although the centroid method was the most consistent method of determination of the condylar axis in separate images that were obtained at different locations through the femoral condyles, as in the study by Murphy et al [22]. Published studies that estimate the mean femoral neck anteversion and to compare this by different methods on the same normal are not widely available. Therefore, no extensive statistical analysis is available on these normal morphological values, especially from the population of Orissa. Other methods, such as ultrasonography and magnetic resonance imaging, exist, but ultrasonography lacks clinical importance because of its inaccuracy [16]. Magnetic resonance imaging has implications in children, in whom the head and neck of the femur is cartilaginous but is associated with limitations of cost and unavailability.

The sample size in our study was small as compared to previous studies; however, considering CT evaluations in normal subjects, the sample size was found to be comparable to that of previous studies. Of the 25 subjects in the study, only six were female; this was because the study sample was purely derived from normal individuals and women in their fifth or sixth decades were unwilling to undergo clinical examination and positioning for the radiological evaluation. Nevertheless, the variation in the sex ratio did not affect the outcome, in validation of the test method because the comparison of different methods was undertaken from values from the same individual. Moreover, the FAV differences between males and females were not significant.

The mean femoral anteversion in the Odisha population was found to be 12.160 ± 4.280(4°-25°), similar to that in the northern part of India (mean 5°-20°); however, this value is lower when compared to the values reported for the Western population [23-24]. In contrast to some studies among the Western population, we did not find any case with retroversion of the femoral head and neck [21]. In the Indian population, the lower value of the anteversion angle could be attributed to more floor-based activities, which require greater external rotation of hip; furthermore, toddlers mostly use the floor for their day-to-day activities and most primary schools make their students sit on the floor during classes.

An analysis of the abovementioned three study methods by ANOVA indicated there was an error between the different methods, as evident from the mean square deviation (MS). Every test may have an unexplained error, but for errors to be significant, the MS value should be a multiple of the unexplained error. In our study, the error between the methods was much higher than what could be attributed to unexplained error. However, this could not specify which method was at variance. Therefore, it is important to more specifically evaluate these methods with the diagnostic value of a test. We found that the diagnostic value of clinical evaluation was greater than that for the radiological method, thus validating its accuracy. Furthermore, in our study, we found that values for the right hip were more accurate than those for the left hip; this occurrence was attributed to the unfamiliar positioning of the examiner when clinically estimating the femoral anteversion of the right hip although this may even be an incidental finding.

## Conclusions

The trochanteric prominence test has accuracy closer to that of CT evaluation when compared to the accuracy of radiological evaluation (Magilligan). This test can be used for screening in population surveys, for approximate estimation in the outpatient department; and in places where the facility of CT evaluation is unavailable.

## References

[1] Brouwer KJ, Molenaar JC, van Linge B (1981). Rotational deformities after femoral shaft fractures in childhood. A retrospective study 27-32 years after the accident. Acta Orthop Scand.

[2] Leonardi F, Rivera F, Zorzan A (2013). Bilateral double osteotomy in severe torsional malalignment syndrome: 16 years follow-up. J Orthop Traumatol.

[3] Folinais D, Thelen P, Delin C, Radiera C, Catonneb Y, Lazennec JY (2013). Measuring femoral and rotational alignment: EOS system versus computed tomography. Orthop Traumatol Surg Res.

[4] Sankar WN, Neubuerger CO, Moseley CF (2009). Femoral anteversion in developmental dysplasia of the hip. J Pediatr Orthop.

[5] Wines AP, McNicol D (2006). Computed tomography measurement of the accuracy of component version in total hip arthroplasty. J Arthroplasty.

[6] Daly PJ, Morrey BF (1992). Operative correction of an unstable total hip arthroplasty. J Bone Joint Surg Am.

[7] Kim HT, Wenger DR (1997). "Functional retroversion" of the femoral head in Legg-Calvé-Perthes disease and epiphyseal dysplasia: analysis of head-neck deformity and its effect on limb position using three-dimensional computed tomography. J Pediatr Orthop.

[8] Gelberman RH, Cohen MS, Shaw BA, Kasser JR, Griffin PP, Wilkinson RH (1986). The association of femoral retroversion with slipped capital femoral epiphysis. J Bone Joint Surg Am.

[9] Stanitski CL, Woo R, Stanitski DF (1996). Femoral version in acute slipped capital femoral epiphysis. J Pediatr Orthop B.

[10] Davids JR, Marshall AD, Blocker ER, Frick SL, Blackhurst DW, Skewes E (2003). Femoral anteversion in children with cerebral palsy. Assessment with two and three-dimensional computed tomography scans. J Bone Joint Surg Am.

[11] Jain AK, Maheswari AV, Singh MP, Nath S, Bhargava SK (2005). Femoral neck anteversion: a comprehensive Indian study. IJO.

[12] Ruwe PA, Gage JR, Ozonoff MB, DeLuca PA (1992). Clinical determination of femoral anteversion. A comparison with established techniques. J Bone Joint Surg Am.

[13] Ogata K, Goldsand EM (1979). A simple biplanar method of measuring femoral anteversion and neck-shaft angle. J Bone Joint Surg Am.

[14] Davids JR, Benfanti P, Blackhurst DW, Allen BL (2002). Assessment of femoral anteversion in children with cerebral palsy: accuracy of the trochanteric prominence angle test. J Pediatr Orthop.

[15] Magilligan DJ (1956). Calculation of the angle of anteversion by means of horizontal lateral roentgenography. JBJS.

[16] Hinderaker T, Uden A, Reikeras O (1994). Direct ultrasonographic measurement of femoral anteversion in newborns. Skeletal Radiol.

[17] Abel MF, Sutherland DH, Wenger DR, Mubarak SJ (1994). Evaluation of CT scans and 3-D reformatted images for quantitative assessment of the hip. J Pediatr Orthop.

[18] Hernandez RJ, Tachdjian MO, Poznanski AK (1981). CT determination of femoral torsion. AJR Am J Roentgenol.

[19] Kuo TY, Skedros JG, Bloebaum RD (2003). Measurement of femoral anteversion by biplane radiography and computed tomography imaging: comparison with an anatomic reference. Invest Radiol.

[20] Brenner DJ, Hall EJ (2007). Computed tomography — an increasing source of radiation exposure. N Engl J Med.

[21] Kingsley PC, Olmsted KL (1948). A study to determine the angle of anteversion of the neck of the femur. J Bone Joint Surg Am.

[22] Murphy SB, Simon SR, Kijewski PK (1987). Femoral anteversion. J Bone Joint Surg Am.

[23] Hubbard DD, Staheli LT (1972). The direct radiographic measurement of femoral torsion using axial tomography: technic comparison with an indirect radiographic method. Clin Orthop Relat Res.

[24] Fabry G, Macewen GD, Shands AR (1973). Torsion of the femur. A follow-up study in normal and abnormal conditions. J Bone Joint Surg Am.

